# Supplementary material for: Spontaneous neurotransmission at evocable synapses predicts their responsiveness to action potentials
Source: Front Cell Neurosci. 2023 Mar 8;17:1129417. doi: 10.3389/fncel.2023.1129417 (PMC10030884; doi:10.3389/fncel.2023.1129417)
Supplement: Supplementary file 1 [file Data_Sheet_1.pdf]

Supplementary Material: Spontaneous neurotransmission at evocable synapses predicts their responsiveness to action potentials

Andreas T. Grasskamp[1], Meida Jusyte[1,2], Anthony W. McCarthy[1], Torsten W.B. Götz[1], Susanne Ditlevsen[3], Alexander M. Walter[1,2,4,*]

[1]Leibniz-Forschungsinstitut für Molekulare Pharmakologie, Berlin, Germany

[2]Einstein Center for Neurosciences, Charité Universitätsmedizin Berlin, Berlin, Germany

[3]Department of Mathematical Sciences, University of Copenhagen, Copenhagen, Denmark

[4]Department of Neuroscience, University of Copenhagen, Copenhagen, Denmark

*Corresponding author: awalter@sund.ku.dk

Department of Neuroscience, University of Copenhagen, Denmark

ORCID: 0000-0002-5895-6529 (ATG) 0000-0001-9948-871X (MJ) 0000-0002-3771-351X (AWM), 0000-0002-5894-1474 (TWBG), 0000-0002-1998-2783 (SD), 0000-0001-5646-4750 (AMW)

Supplementary Figures

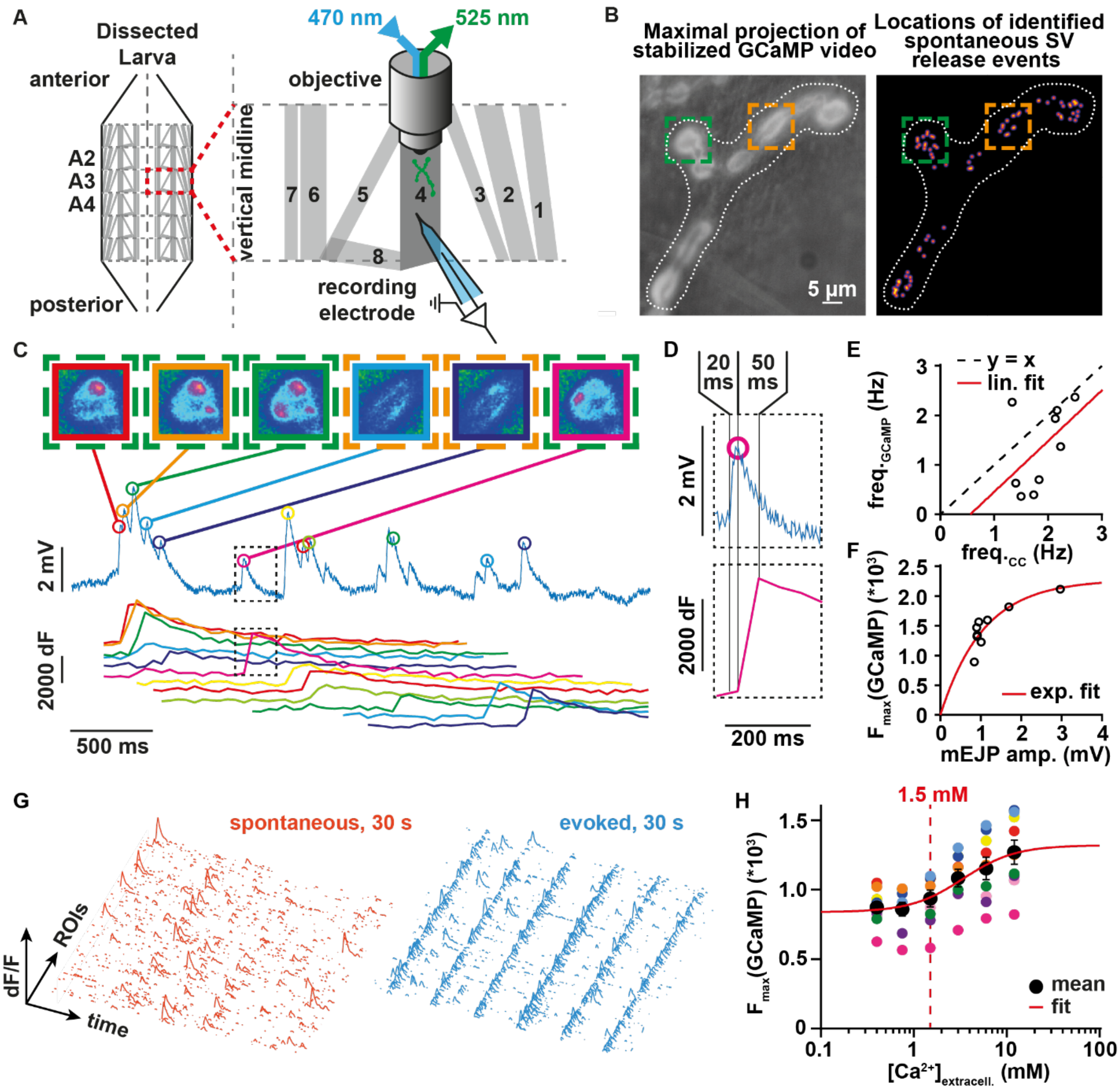

Supplementary Figure 1. Further characterization of optical signals and their relation to synaptic activity. (**A**) Scheme of the experimental setup; current clamp recordings and GCaMP fluorescence measurements are performed in the same muscle 4 NMJ. (**B**) Spontaneous event detection in GCaMP fluorescence assay independent throughout the NMJ (see Methods & supplementary Figure 2 for further details). Two ROIs over active boutons are marked in green and orange. (**C**) Events detected in current-clamp recordings and GCaMP fluorescence assay coincide to a large degree. Spontaneous events in bouton ROIs marked in **B** are observable with high spatial and temporal resolution (**D**) Blow-up of black ROI in **C** to show details of the temporal relation of current-clamp (top) and fluorescence (bottom) measurement. (**E**) Animal-wise (N = 9 cells&animals) spontaneous event frequencies measured in current-clamp recordings plotted against frequencies measured in fluorescence recordings. Linear fit on cell means in red, dashed black line represents x = y. (**F**) Animal-wise (N = 9 cells&animals) mEJP amplitudes measured in current-clamp plotted against maximal fluorescence amplitudes measured in fluorescence assay. Exponential fit on cell means in red. (**G**) 3D representation of 30 s of spontaneous (orange) and AP-evoked (blue) event

amplitudes over all ROIs in one NMJ (**H**) Quantification of spontaneous event amplitudes over six extracellular Ca^{2+} concentrations (0.4, 0.75, 1.5, 3, 6, 12 mM) shows no saturation at physiological 1.5 mM $[Ca^{2+}]_{ext.}$ (N = 9 animals). Red line represents hill curve fit on individual values. Best fit parameters (compare equation 7 in manuscript): $F_{max} = 481.8$; $h = 1.543$; $K_A = 3.393$; $C = 838.8$. Data is shown as mean amplitude per animal (colored dots) or animal-wise mean±SEM (black). Scale bar in **B**: 5 µm.

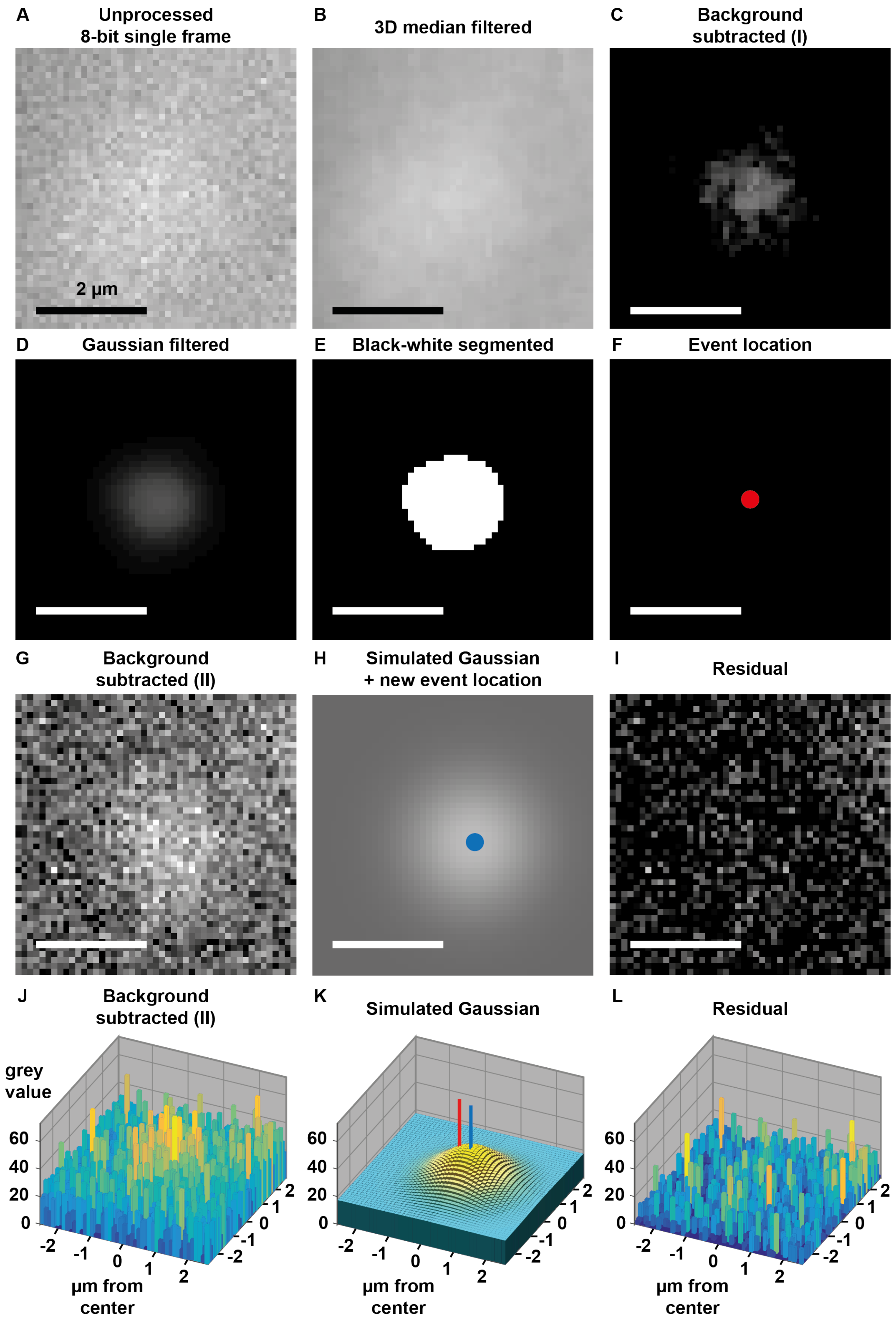

Supplementary Figure 2. Sequence of event detection algorithm for spontaneous activity throughout the NMJ. (A) 47x47 pixel cutout from the original, 8-bit video showing a typical spontaneous event. **(B)** Image from A after 3D median filtering for noise reduction. **(C)** Image from **B** after subtraction of brightest

features of the 10th through 6th preceding frames. **(D)** Image from C after application of a Gaussian filter for noise reduction. **I** Image from **D** segmented into grey values below or equal to 2 (black) or greater (white). **(F)** Determined location of the event. **(G)** Maximum projection of 6 frames from original video **A** after subtraction of average features of the 10th through 6th preceding frames. **(H)** 2D Gaussian fit to **G**. **(I)** Residual of Gaussian fit: Result of subtracting **H** from **G**. **(J)-(L)** 3D representations of images in **G-I**. Scale bar: 2 μm.

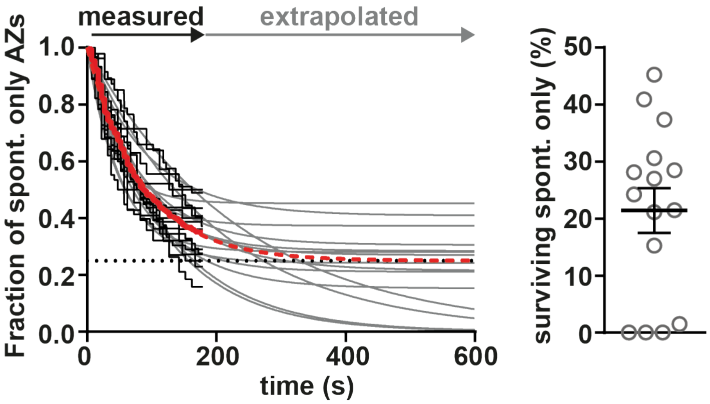

Supplementary Figure 3. The majority of BRP-positive AZs are AP-responsive, but a minority is dedicated to spontaneous transmission. Left: Cell-wise (N =15 animals) survival analysis: AZs found spontaneously active during the first recording episode (1 in Fig. 2A) are tested for their "survival" as "spontaneous only" AZs in the second recording episode (2 in Fig. 2A) where 36 APs are administered at 0.2 Hz. Once a previously spontaneously active AZ responds to an AP, this is considered the "death" of a "spontaneous only" AZ. A fraction of 1 represents all AZs spontaneously active before AP application. Individual black lines indicate the behavior each of the investigated animals/NMJs, the grey lines are fits with a mono exponential decay function that includes a plateau value (see methods for details). The Red line is the fit to the average behavior of all investigated NMJs (N = 15 animals). Right: Fraction of exclusively spontaneously active AZs in relation to all spontaneously active AZs (plateau values from the graph depicted on the left). Circles represent the individual plateau values (fraction of AZs dedicated to spontaneous transmission only) in each of the 15 animals, the vertical line and error bars indicate the mean and SEM. A model with plateau was preferred based on Akaike's Information criterion (see methods for details).

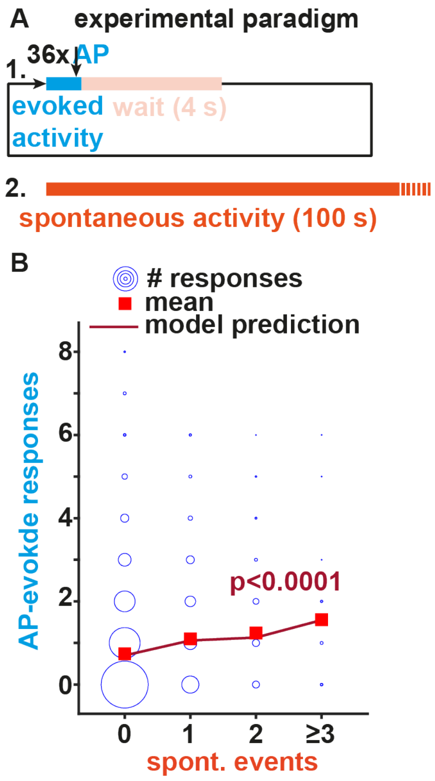

Supplementary Figure 4. Inverse experiment where AP-evoked activity is read out before spontaneous activity. (**A**) Experimental paradigm. 36 APs are elicited at 0.2 Hz (blue) before spontaneous events (orange) are recorded for 100 s in isolation. (**B**) Analysis of the relation between the observed AP-evoked and spontaneous transmission events at individual BRP-positive AZs from 22 animals. Events from all imaged AZs in the 22 animals are pooled. The size of the circles relates to the number of observations (between 1 and 1493). Red squares indicate mean number of evoked responses, the line indicates the model prediction (see methods). Number of BRP-positive AZs investigated: n(AZs)=3194, number of animals in as a function of spontaneous activity: N(0)=22, N(1)=21, N(2)=19, N(≥3)=9).

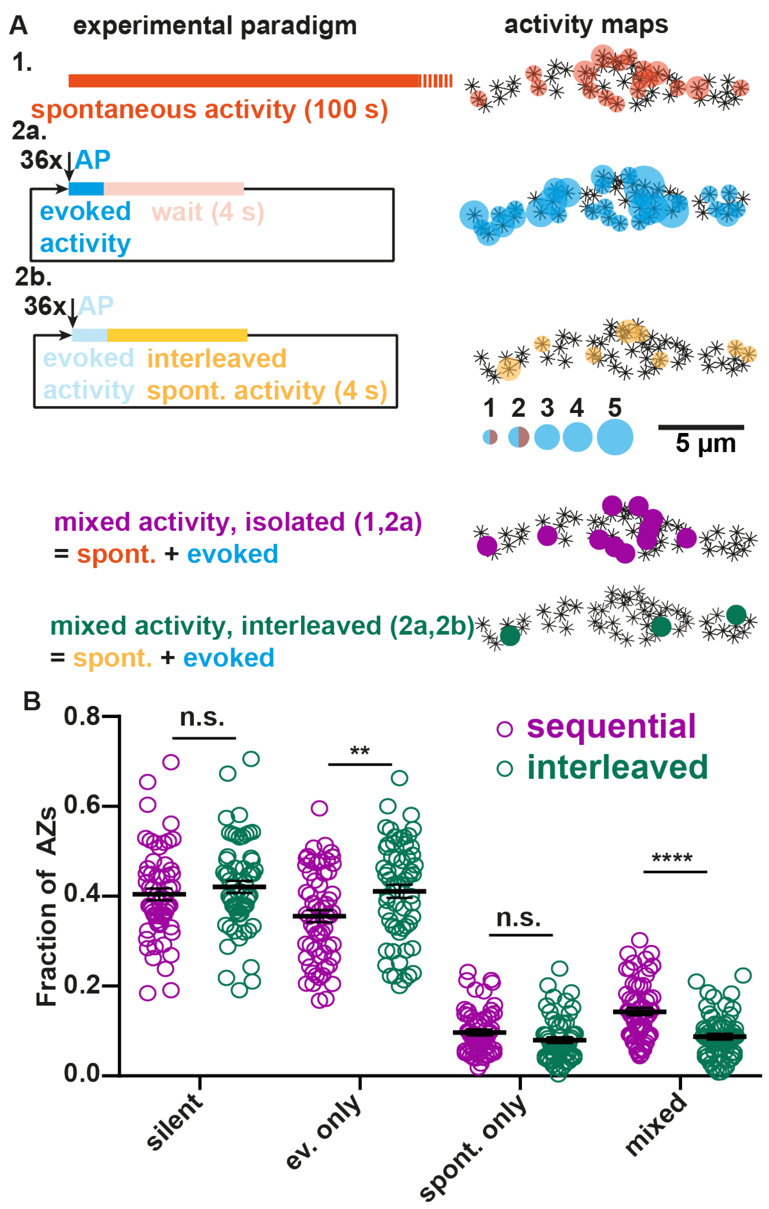

Supplementary Figure 5. Spontaneous activity at BRP-positive AZs is reduced when sampled in-between AP stimuli. (**A**) Left: Two experimental designs to assess spontaneous activity, either in isolation by first sampling spontaneous and then AP-evoked activity (1&2a) or interleaved between the AP-stimuli (2a&2b). Right: Activity maps for isolated spontaneous events (from episode 1), AP-evoked events (from episode 2a) and interleaved spontaneous events (from episode 2b). The same AP-evoked activity (from episode 2a) is used for comparison with the isolated (1) and interleaved (2b) spontaneous activity. (**B**) Animal-wise quantification (N = 59 animals) of fractions of AZs active in the four activity categories (no activity/silent, only AP-evoked activity observed, only spontaneous activity observed, or both activities observed) either measurement sequentially in isolation (purple) or interleaved (green). Lines/Error bars indicate mean and SEM. * <0.05; ** p<0.01; ***p<0.001; ****p<0.0001; n.s. not significant in a two-tailed Student's t-test.

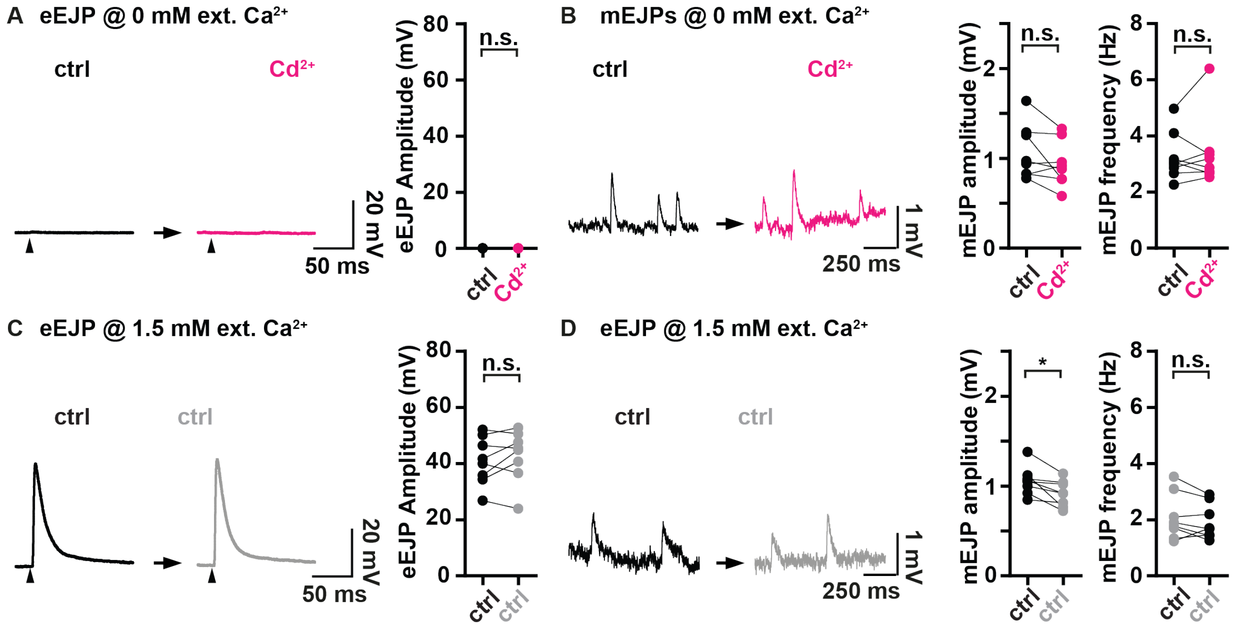

Supplementary Figure 6. Application of Cd^{2+} in the absence of extracellular Ca^{2+} has no effects on mEJPs and their frequency is not reduced by the recording paradigm itself. (**A&B**) Assessment of the consequences of Cd^{2+}-application (300 µM, black→magenta) on AP-evoked eEJPs (A) and on spontaneous miniature excitatory junction potentials (mEJPs) (B) in paired current clamp recordings of muscle 4 NMJs in the absence of extracellular Ca^{2+} (no Ca^{2+} was added to the extracellular solution and 2 mM EGTA was added to buffer any free remaining Ca^{2+}). (**A**) Left: Representative eEJP before (black) and after (magenta) application of 300 µM $CdCl_2$. Right: Animal-wise quantification of eEJP amplitudes before (black) and after (magenta) Cd^{2+} application. (**B**) Left: Representative mEJP traces before (black) and after (magenta) Cd^{2+} application. Right: Cell-wise quantification of mEJP amplitudes and -frequencies. (**C&D**) Assessment of the consequences of the experimental paradigm (no treatment, black→grey) on AP-evoked eEJPs (C) and on spontaneous mEJPs (D) in paired current clamp recordings of muscle 4 NMJs in the presence of 1.5 mM Ca^{2+} in the extracellular solution (same condition as in Fig. 3C&D). (**C**) Left: Representative eEJP before (black) and after (grey) application of a mock solution. Right: Animal-wise quantification of eEJP amplitudes before (black) and after (grey) application of a mock solution. (**D**) Left: Representative mEJP traces before (black) and after (grey) application of a mock solution. Right: Cell-wise quantification of mEJP amplitudes and -frequencies. Arrowheads indicate time of stimulation. Number of animals in (A&B and C&D): N=8. N.s. not significant; * $p<0.05$; *** $p<0.001$; **** $p<0.0001$. Paired parametric t-test for comparisons in (A&B and C&D).

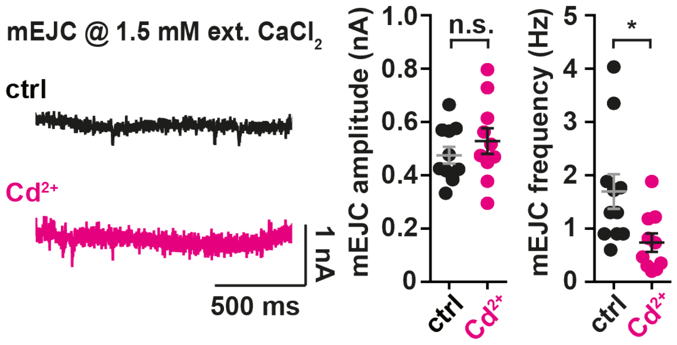

Supplementary Figure 7. Investigation of spontaneous transmission upon application of the voltage-gated calcium channel blocker Cd^{2+} in voltage clamp recordings. Left: Representative miniature excitatory junction current (mEJC) traces of ctrl cells (black) or cells treated with 740.7 µM Cd^{2+} (magenta) recorded in the presence of 1.5 mM extracellular Ca^{2+}. Right: Cell-wise quantification of mEJC amplitudes and mEJC frequencies. These experiments were recorded in parallel to the GCaMP Experiments of Figure 3B. This is the reason why the same Cd^{2+} concentration was used and that the comparison is between groups of animals with and without treatment. Number of animals: N(ctrl) = 11, N(Cd^{2+}) = 10. Horizontal bars indicate mean, error bars SEM. n.s. not significant; * $p<0.05$. Two-tailed parametric Student's t-test.

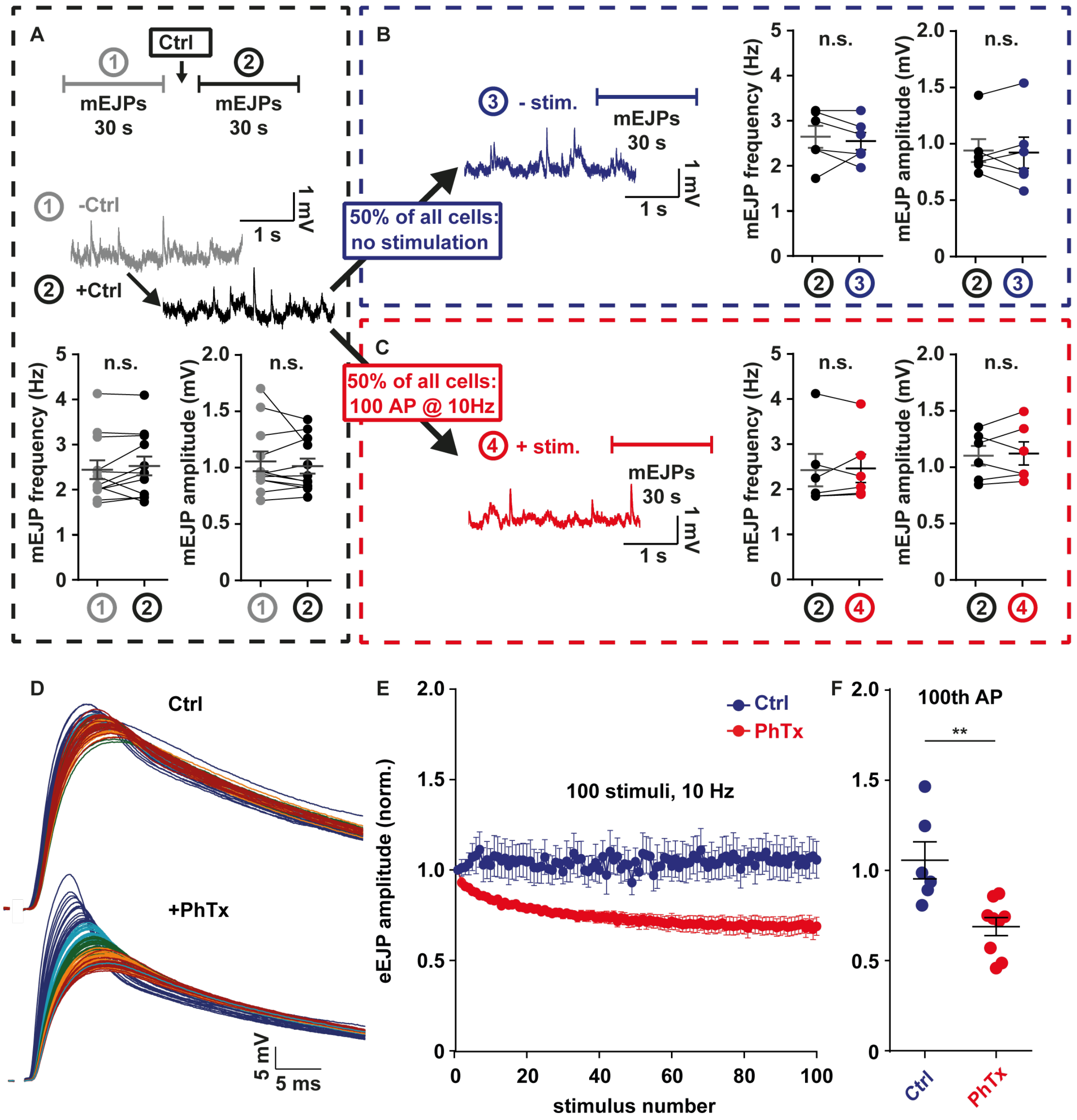

Supplementary Figure 8. (A-C) Analogous to Figure 4, testing whether AP stimulation alone affects spontaneous neurotransmission **D-F**; (**A**) mEJP frequency and amplitude quantification before (grey) and after (black) control treatment (N = 12 animals) (**B**) mEJP frequency and amplitude quantification after control treatment and without stimulation (N = 6 animals) (**C**) mEJP frequency and amplitude quantification after stimulation (10 s wait; N = 6 animals). (**D**) Influence of stimulation on eEJP amplitude over 100 stimuli applied at 10 Hz; top: control conditions, only minor deterioration of amplitudes over 100 APs; bottom: +PhTx, marked decrease of amplitudes over 100 APs. Representative traces shown from 1st eEJP amplitude (blue) to 100th eEJP amplitude (red) (**E**) Sequential quantification of eEJP amplitudes over 100 stimuli applied at 10 Hz in either control (ctrl, blue) or +PhTx (red, 4 µM PhTx) treatment. (**F**) Quantification of 100th eEJP amplitudes in ctrl (blue, N=6 animals) or PhTx conditions (red, N=9 animals). **p<0.01; n.s. not significant. Error bars indicate SEM.

Supplementary data and statistics

Figure	Panel	Group	Measure	Mean	SEM	Significance level/alpha	Test type	N (animals)
**Supp. Fig. 1**	H	0.4 mM $[Ca^{2+2+}]_{ext}$	mean GCaMP5G spontaneous event amplitude	873.390	42.670			9
		0.75 mM $[Ca^{2+2+}]_{ext}$		859.783	49.161			
		1.5 mM $[Ca^{2+2+}]_{ext}$		936.153	60.632			
		3 mM $[Ca^{2+2+}]_{ext}$		1084.005	62.637			
		6 mM $[Ca^{2+2+}]_{ext}$		1152.955	80.846			
		12 mM $[Ca^{2+2+}]_{ext}$		1270.339	87.833			

Table 1 – Data relating to Supplementary Figure 1

Figure	Measure	Mean	SEM
**Supp. Fig. 3**	mean of exp. fit plateaus	0.2498	0.001966
**Supp. Fig. 3**	percentage of surviving spont. only Azs	0.2142	0.03192

Table 2 – Data relating to Supplementary Figure 3

Figure	Panel	Measur<br>e	Test type	ANOVA summary		Mutliple comparisons	Mean 1	Mean 1	Mean Diff.	SE of Diff	N1	N2	Adjusted P Value
**2**	B	Fractio<br>n of<br>AP-<br>respons<br>ive AZs	ANOVA	F	11.9	0 vs. 1	0 vs. 1	0.467	-0.1146	0.0407<br>2	59	59	0.0273
				P value	<0.0001	0 vs. 2	0 vs. 2	0.467	-0.1752	0.0409	59	58	0.0002
				P value summary	****	0 vs. >2	0 vs. >2	0.467	-0.2789	0.0501<br>6	59	29	<0.0001
				Significant diff. Among means (P > 0.05)	Yes	1 vs. 2	1 vs. 2	0.5817	-<br>0.0605<br>9	0.0409	59	58	0.4506
				R square	0.1518	1 vs. >2	1 vs. >2	0.5817	-0.1643	0.0501<br>6	59	29	0.0068
				Number of values	205	2 vs. >2	2 vs. >2	0.6423	-0.1037	0.0503	58	29	0.1693
				Number of treatments	4								

Table 3 – Data relating to Figure 2B

counts	0 spont	1 spont	2 spont	>2 spont
**0 ev**	3966	696	144	14
**1 ev**	1943	490	122	19
**2 ev**	890	278	66	7
**3 ev**	401	148	33	7
**4 ev**	180	55	26	6
**5 ev**	78	30	12	2
**6 ev**	24	21	3	1
**7 ev**	8	2	1	1
**8 ev**	2	1	0	0
**Mean evoked activity**	0.83	1.17	1.33	1.77
**Fitted mean evoked activity**	0.78	1.1	1.27	1.78

Table 4 – Data relating to Figure 2C

counts	0 spont	1 spont	2 spont	>2 spont
**0 ev**	1493	199	33	5
**1 ev**	643	116	34	6
**2 ev**	293	76	23	4
**3 ev**	110	43	6	1
**4 ev**	45	13	3	0
**5 ev**	20	6	2	1
**6 ev**	5	4	1	1
**7 ev**	6	0	0	0
**8 ev**	2	0	0	0
**Mean evoked activity**	0.74	1.10	1.24	1.56
**Fitted mean evoked activity**	0.7	1.06	1.13	1.55

Table 5 – Data relating to Supplementary Figure 4 panel B

Figure	Panel	Measure	Group	Mean	SEM	P value	Test type	N (animals)	t	df	F
**Supp. Fig. 5**	B	fraction of all Azs	silent, sequential	0.4042	0.01329	0.3711	Unpaired t test	59	0.8979	116	1.016
			silent, interleaved	0.4211	0.01340						
			ev only, sequential	0.3556	0.01345	0.0061			2.794	116	1.193
			ev only, interleaved	0.4113	0.01469						
			sp only, sequential	0.09686	0.006667	0.0704			1.826	116	1.066
			sp only, interleaved	0.07991	0.006456						
			mixed, sequential	0.1433	0.008586	<0.0001			5.169	116	1.742
			mixed, interleaved	0.8765	0.006504						

Table 6 – Data relating to Supplementary Figure 5 panel B

Figure	Measure	Group	Mean	SEM	Significance level/alpha	Test type	Comment	N (animals)
**Fig. 3A**	average GCaMP fluorescence amplitude	ctrl	371.7	20.18	0.5573	unpaired non-parametric t-test		18
		TTX	353.5	20.45				
	avg. spont. event frequency (Hz/AZ)	ctrl	0.00217	0.0002246	0.7123	unpaired non-parametric t-test		
		TTX	0.002162	0.0003297				
**Fig. 3B**	average GCaMP fluorescence amplitude	ctrl	409.4	35.19	0.0668	unpaired non-parametric t-test		11
		Cd	330.9	18.01			2 animals showed no events	9
	avg. spont. event frequency (Hz/AZ)	ctrl	0.00221	0.000354	< 0.0001	unpaired non-parametric t-test		11
		Cd	0.0002248	0.00007827				11

Table 7 – Data relating to Figure 3A,B

Figure	Measure	Group	Mean	SEM	Significance level/alpha	Test type	N (animals)
**Fig. 3C**	eEJP amplitude (mV), 1.5 mM $[Ca^{2+}]_{ext.}$	ctrl before	51.73	2.242	<0.0001	paired parametric t-test	8
		$CdCl_2$	0	0			
**Fig. 3D**	mEJP amplitude (mV), 1.5 mM $[Ca^{2+}]_{ext.}$	ctrl before	1.046	0.07287	<0.0001	paired parametric t-test	8
		$CdCl_2$	0.7458	0.05095			
	mEJP frequency(Hz), 1.5 mM $[Ca^{2+}]_{ext.}$	ctrl before	3.013	0.5100	0.0068	paired parametric t-test	8
		$CdCl_2$	2.250	0.4348			

Table 8 – Data relating to Figure 3C,D

Figure	Panel	Measure	Group	Mean	SEM	Significance level/alpha	Test type	Comment	N (animals)
**Supp. Fig. 6**	A	eEJP amplitude (mV), 0 mM $[Ca^{2+}]_{ext.}$	ctrl before	0	0			no test could be performed since all values were 0	8
			$CdCl_2$	0	0				
	C	eEJP amplitude (mV), 1.5 mM $[Ca^{2+}]_{ext.}$	ctrl before	40.94	3.030	0.2986	paired parametric t-test		
			ctrl after	42.83	3.264				
	D	mEJP amplitude (mV), 1.5 mM $[Ca^{2+}]_{ext.}$	ctrl before	1.057	0.05586	0.0121	paired parametric t-test		8
			ctrl after	0.9178	0.05121				
	B	mEJP amplitude (mV), 0 mM $[Ca^{2+}]_{ext.}$	ctrl before	0.9713	0.1602	0.1467	paired parametric t-test		8
			ctrl after	0.8994	0.1329				
	D	mEJP frequency(Hz), 1.5 mM $[Ca^{2+}]_{ext.}$	ctrl before	2.083	0.2898	0.2543	paired parametric t-test		8
			ctrl after	1.925	0.2211				
	B	mEJP frequency (Hz), 0 mM $[Ca^{2+}]_{ext.}$	ctrl before	2.421	0.1776	0.4166	paired parametric t-test		8
			ctrl after	2.508	0.2175				

Table 9 – Data relating to Supplementary Figure 6

Figure	Measure	Group	Mean	SEM	Significance level/alpha	Test type	Comment	N (animals)
**Supp. Fig. 7**	mEJC amplitude (nA)	ctrl	0.4754	0.03126	0.3618	unpaired parametric t-test		11
		Cd	0.5282	0.04835				10
	mEJC frequency (Hz)	ctrl	1.695	0.3255	0.0209	unpaired parametric t-test		11
		Cd	0.7383	0.1734				10

Table 10 – Data relating to supplementary Figure 7

Figure	Panel	Measure	Group	Mean	SEM	Significance evel/alpha	Test type	Comment	N (animals)
**Fig. 4**	D	mEJP frequency (Hz)	before PhTx	2.417	0.1329	0.0435	paired parametric t-test		18
		mEJP frequency (Hz)	after PhTx	2.226	0.1917				18
	D	mEJP amplitude (mV)	before PhTx	0.8902	0.03051	< 0.0001	paired parametric t-test		18
		mEJP amplitude (mV)	after PhTx	0.5702	0.02297				18
	E	mEJP frequency (Hz)	after PhTx	2.163	0.2705	0.8962	paired parametric t-test		9
		mEJP frequency (Hz)	no stim	2.144	0.2429				9
	E	mEJP amplitude (mV)	after PhTx	0.5261	0.03291	0.8328	paired parametric t-test		9
		mEJP amplitude (mV)	no stim	0.5218	0.02073				9
	F	mEJP frequency (Hz)	after PhTx	2.289	0.2863	0.0027	paired parametric t-test		9
		mEJP frequency (Hz)	stim	1.726	0.179				9
	F	mEJP amplitude (mV)	after PhTx	0.6144	0.02594	0.2031 / 0.1371	Wilcoxon matched-pairs signed rank test / paired parametric t-test	Group “after PhTx” matched with “stimulation” group failed D’Agostino & Pearson omnibus normality test	9
		mEJP amplitude (mV)	stim	0.5675	0.04475				9

Table 11 – Data relating to Figure 4

Figure	Panel	Measure	Group	Mean	SEM	Significance evel/alpha	Test type	Comment	N (animals)
Supp. Fig. 8	A	mEJP frequency (Hz)	before ctrl	2.444	0.2086	0.3491	paired nonparametric t-test		12
		mEJP frequency (Hz)	after ctrl	2.528	0.2091				12
	A	mEJP amplitude (mV)	before ctrl	1.057	0.08722	0.5186	paired nonparametric t-test		12
		mEJP amplitude (mV)	after ctrl	1.014	0.06722				12
	B	mEJP frequency (Hz)	after ctrl	2.650	0.2426	0.6250	paired nonparametric t-test		6
		mEJP frequency (Hz)	after ctrl, -stim	2.556	0.1891				6
	B	mEJP amplitude (mV)	after ctrl	0.9385	0.1011	0.8438	paired nonparametric t-test		6
		mEJP amplitude (mV)	after ctrl, -stim	0.9202	0.1380				6
	C	mEJP frequency (Hz)	after ctrl	2.406	0.3572	0.8438	paired nonparametric t-test		6
		mEJP frequency (Hz)	after ctrl, +stim	2.444	0.3129				6
	C	mEJP amplitude (mV)	after ctrl	1.090	0.08576	0.6875	paired nonparametric t-test		6
		mEJP amplitude (mV)	after ctrl, +stim	1.109	0.1024				6
	F	norm. eEJP amplitude	ctrl	1.057	0.1020	0.0016/0.0033	Mann-Whitney U test/unpaired parametric t-test		6
			PhTx	0.6888	0.04986				9

Table 12 – Data relating to Supplementary Figure 8
